# Bridging Materials and Analytics: A Comprehensive Review of Characterization Approaches in Metal-Based Solid-State Hydrogen Storage

**DOI:** 10.3390/molecules29215014

**Published:** 2024-10-23

**Authors:** Yaohui Xu, Yang Zhou, Yuting Li, Yang Zheng

**Affiliations:** 1 Laboratory for Functional Materials, School of New Energy Materials and Chemistry, Leshan Normal University, Leshan 614000, China; 2 State Key Laboratory of New Textile Materials and Advanced Processing Technology, School of Textile Science and Engineering, Wuhan Textile University, Wuhan 430200, China; 3 Leshan West Silicon Materials Photovoltaic New Energy Industry Technology Research Institute, Leshan 614000, China; 4 College of Materials Science and Engineering, National Engineering Research Center for Magnesium Alloys, Chongqing University, Chongqing 400044, China; 5 The State Key Laboratory of Refractories and Metallurgy, Institute of Advanced Materials and Nanotechnology, Wuhan University of Science and Technology, Wuhan 430081, China

**Keywords:** solid-state hydrogen storage materials, characterization techniques, hydrogen storage performance, structure and composition

## Abstract

The advancement of solid-state hydrogen storage materials is critical for the realization of a sustainable hydrogen economy. This comprehensive review elucidates the state-of-the-art characterization techniques employed in solid-state hydrogen storage research, emphasizing their principles, advantages, limitations, and synergistic applications. We critically analyze conventional methods such as the Sieverts technique, gravimetric analysis, and secondary ion mass spectrometry (SIMS), alongside composite and structure approaches including Raman spectroscopy, X-ray diffraction (XRD), X-ray photoelectron spectroscopy (XPS), scanning electron microscopy (SEM), transmission electron microscopy (TEM), and atomic force microscopy (AFM). This review highlights the crucial role of in situ and operando characterization in unraveling the complex mechanisms of hydrogen sorption and desorption. We address the challenges associated with characterizing metal-based solid-state hydrogen storage materials discussing innovative strategies to overcome these obstacles. Furthermore, we explore the integration of advanced computational modeling and data-driven approaches with experimental techniques to enhance our understanding of hydrogen–material interactions at the atomic and molecular levels. This paper also provides a critical assessment of the practical considerations in characterization, including equipment accessibility, sample preparation protocols, and cost-effectiveness. By synthesizing recent advancements and identifying key research directions, this review aims to guide future efforts in the development and optimization of high-performance solid-state hydrogen storage materials, ultimately contributing to the broader goal of sustainable energy systems.

## 1. Introduction

The quest for clean and sustainable energy solutions has propelled the development of solid-state hydrogen storage materials to the forefront of scientific research [1,2,3]. These materials offer several advantages over traditional compressed or liquefied hydrogen storage methods, including higher storage capacities, improved safety, and ease of handling [4,5,6]. However, the complex nature of hydrogen storage mechanisms and the diverse range of materials being investigated present significant challenges in understanding their behavior and optimizing their performance [7,8,9].

To advance solid-state hydrogen storage materials from laboratory research to practical application, a comprehensive understanding of the microstructure, compositional changes, and dynamic behavior during hydrogenation/dehydrogenation processes is essential, and this is precisely where characterization techniques play a critical role [10,11]. For instance, techniques like the Sieverts method and gravimetric analysis are excellent for measuring the hydrogen storage capacity and kinetic behavior of materials, but when it comes to analyzing microstructural changes, they need to be complemented by optical and surface analysis techniques such as X-ray diffraction (XRD), Raman spectroscopy, and X-ray photoelectron spectroscopy (XPS). Additionally, neutron scattering is advantageous for studying the distribution and dynamic behavior of hydrogen atoms, while electrochemical methods are particularly effective in analyzing the electronic structure and surface reactivity of materials [12,13,14,15,16,17,18].

In recent years, with the continuous advancement of materials science, metal-based hydrogen storage materials have become a research hotspot due to their unique structures and excellent hydrogen storage performance [19,20,21,22]. These novel materials demonstrate outstanding potential for hydrogen storage, particularly in terms of increasing storage density and adsorption/desorption rates. However, the structural and performance complexity of these materials presents new challenges for characterization [23,24,25,26]. Traditional characterization techniques have certain limitations in terms of precision, sensitivity, and applicability, making it difficult to fully reveal the dynamic behavior of these new materials during the hydrogen storage process [27]. It is important to note that there is typically no “optimal” technique when selecting and applying characterization methods. Each technique has its strengths in revealing specific properties of materials, but they are also accompanied by limitations [28,29,30]. Thus, different characterization methods often complement and synergize with one another.

In summary, characterization techniques for solid-state hydrogen storage materials are key tools for understanding and optimizing their performance. We have conducted a comprehensive review of various characterization methods, aiming to provide researchers with a thorough technical reference that encompasses both traditional and emerging methods. By comparing the advantages and limitations of different techniques in their applications, we emphasize their complementarity and synergistic relationships, rather than seeking the so-called “optimal” testing method. Each characterization technique has its unique applicable scenarios and focal points, and no single method can fully elucidate all the mechanisms involved in hydrogen storage within solid-state materials. Therefore, this review particularly focuses on the unique demands of characterizing these novel materials and discusses how existing techniques can be improved or new methods developed to address these challenges. This review not only provides researchers with a comprehensive technical overview of the field of solid-state hydrogen storage characterization but also highlights the difficulties in characterizing emerging materials and the trends for future technological development. Through an in-depth analysis of various characterization techniques, we hope to provide a theoretical basis and technical support for achieving efficient and sustainable hydrogen storage solutions, thereby contributing to the continued development and application of clean energy technologies.

## 2. Hydrogen Storage Performance Characterization Techniques

Hydrogen storage materials are crucial for advancing hydrogen-based energy systems, and their performance is highly dependent on their physical and chemical properties [31]. To thoroughly understand these materials, several fundamental characterization techniques are employed. The Sieverts method, gravimetric analysis, secondary ion mass spectrometry, neutron scattering, and electrochemical methods represent some of the most essential tools in this domain. By leveraging these methods, researchers can systematically evaluate and optimize hydrogen storage materials to meet the demands of practical applications in energy storage and conversion. Table 1 briefly summarizes the pros and cons of each basic characterization technique.

### 2.1. Sieverts Method

**Figure 1:**
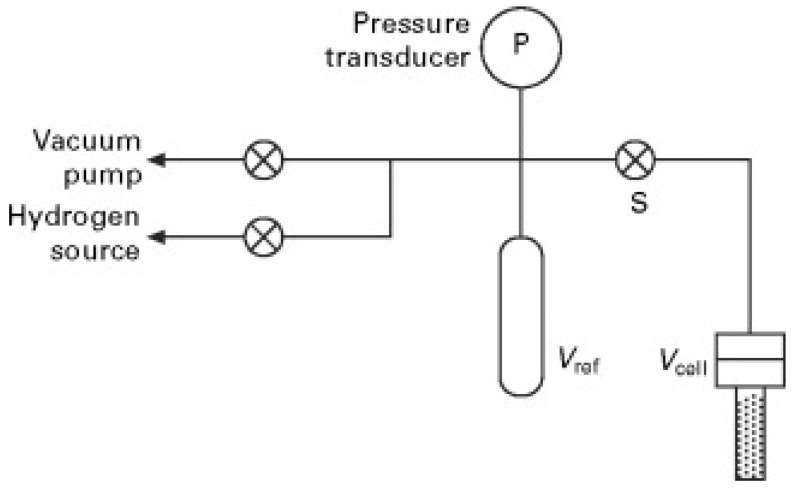
Schematic representation of a Sieverts apparatus [34].

The Sieverts method, also known as the volumetric method, is one of the most widely used techniques for characterizing hydrogen storage materials [32,33]. The core principle is based on the ideal gas law and the pressure changes during the gas absorption and desorption processes. The simplified schematic of the device is shown in Figure 1 [34]. When high-pressure gas comes into contact with a metal sample, the gas dissolves into the metal. According to Henry’s law, the solubility of the gas in the metal is proportional to the partial pressure of the gas. By measuring the pressure changes before and after gas injection with a high-precision pressure sensor system, the amount of gas dissolved in the metal sample can be calculated.

The Sieverts method offers several advantages, including simplicity, reliability, and the ability to measure hydrogen sorption isotherms over a wide range of pressures and temperatures [35]. It provides essential thermodynamic information, such as the equilibrium pressure, enthalpy, and entropy of the hydrogen sorption reactions [36]. To overcome the limitations of the Sieverts method, researchers have developed advanced apparatus with improved temperature control, high-precision pressure sensors, and automated data acquisition systems. The Sieverts method is widely utilized in mainstream hydrogen testing and analysis instruments for analyzing hydrogen storage density in hydrogen storage materials. This technique provides essential measurements of hydrogen absorption and desorption capacities, critical for evaluating material performance in hydrogen storage applications [26,30]. Rigorous calibration procedures and error analysis methods have been implemented to enhance the accuracy and reliability of the measurements. The Sieverts method is particularly crucial for evaluating the hydrogen storage capacity and kinetics of metal hydrides.

**Figure 2:**
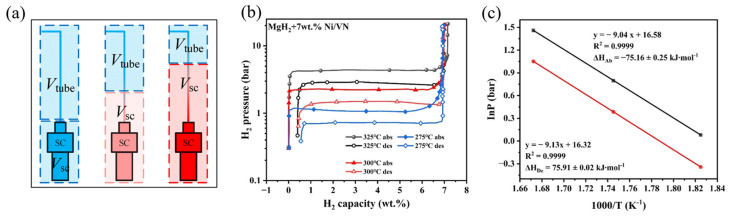
(**a**) Diagram of temperature gradients in the reactor [38]. The MgH_2_ + 7 wt.% Ni/VN (**b**) PCT curves at 325 and 275 °C and (**c**) Van’t Hoff plots [72].

Existing volumetric measurement instruments often suffer from low efficiency due to insufficient calibration techniques, temperature gradients, and limited automation. As shown in Figure 2a, the curve changes with the temperature of the sample. As the sample temperature increases, the line moves further from the sample cell, resulting in an increase in the apparent volume of the sample cell and a decrease in the apparent volume of the tube. The position of this line can be determined through calibration methods and is defined as a function of temperature. Zhu et al. [38] proposed a novel volumetric calibration and thermal gradient resistance method by introducing a continuous function to overcome temperature gradients across the entire test temperature range. This method was validated through TPD/TPA tests on MgH_2_ powder, demonstrating automatic temperature control. Moreover, the new method enabled the completion of three PCT curves within 1.5 days. This innovative approach has the potential to make Sieverts instruments more effective, accurate, and reliable tools for characterizing hydrogen storage materials.

The PCT curve is primarily used to describe the relationship between the composition of a chemical system and pressure under isothermal conditions, and it is commonly applied in the study of gas adsorption and metal hydrides for hydrogen storage. By analyzing the PCT curve, insights can be gained into the phase behavior of the system, adsorption or desorption processes, and the hydrogen storage capacity of the material. The van’t Hoff equation *lnK* = −Δ*H/RT* + Δ*S/R*, where *K* is the equilibrium constant, Δ*H* is the standard reaction enthalpy (kJ/mol), Δ*S* is the standard reaction entropy (J/mol·K), *R* is the gas constant (J/mol·K), and *T* is the temperature (K), reveals the relationship between the chemical equilibrium constant and temperature, forming the theoretical foundation for studying the effect of temperature on equilibrium in reaction thermodynamics. Wu et al. [72] measured the PCT curves of MgH_2_-7 wt.% Ni/VN and ball-milled MgH_2_ samples at different temperatures (Figure 2b). The results show that the plateau pressures for MgH_2_-7 wt.% Ni/VN during desorption at 548, 573, and 598 K were 0.72, 1.49, and 2.90 bar, respectively, and during absorption, the plateau pressures were 1.10, 2.25, and 4.20 bar, respectively. For ball-milled MgH_2_, the desorption plateau pressures at 598, 623, and 648 K were 2.20, 4.12, and 7.40 bar, respectively, while the absorption pressures were 3.82, 7.00, and 12.20 bar. Using the van’t Hoff equation to calculate the ΔH for hydrogen absorption and desorption of MgH_2_-7 wt.% Ni/VN at different temperatures based on the plateau pressures, the values were found to be −75.16 ± 0.25 and 75.91 ± 0.02 kJ·mol^−1^ (Figure 2c), indicating no significant reduction compared to pure MgH_2_.

By experimentally determining the changes in the equilibrium constant with temperature, thermodynamic parameters such as enthalpy and entropy changes can be obtained. The van’t Hoff equation not only helps in understanding the influence of temperature on the direction and extent of chemical reactions but also has wide applications in material design, catalysis, and industrial process optimization. The combination of the PCT curve and the van’t Hoff equation allows for in-depth study of thermodynamic behavior in multi-phase systems such as gas–solid and liquid–solid phases, providing reliable thermodynamic parameters to optimize material performance and understand reaction mechanisms.

### 2.2. Gravimetric Analysis

Gravimetric analysis is another fundamental technique for characterizing hydrogen storage materials. It involves measuring the mass change of a sample during hydrogen absorption or desorption processes [39]. One of the main advantages of gravimetric analysis is its high sensitivity, allowing for the detection of small mass changes associated with hydrogen sorption. It provides real-time monitoring of the hydrogen uptake and release, enabling the study of kinetics and the determination of the rate-limiting steps in the sorption processes [40]. Gravimetric analysis offers unique advantages in studying hydrogen storage materials, particularly for understanding sorption kinetics and cycling stability. However, due to the high cost of high-temperature magnetic levitation balances, this method is rarely used in the practical analysis of hydrogen storage materials.

**Figure 3:**
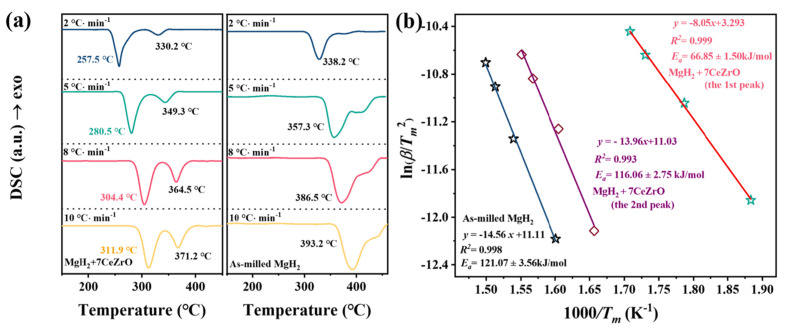
(**a**) The DSC profiles at 2, 5, 8, and 10 °C·min^−1^ of MgH_2_-7CeZrO and ball-milled MgH_2_; (**b**) the apparent dehydrogenation activation energy of MgH_2_-7CeZrO and undoped MgH_2_ by Kissinger equation.

Thermogravimetric analysis (TG) is a method used to measure changes in the mass of a sample as the temperature increases or under isothermal conditions [42,44]. TG is usually combined with differential scanning calorimetry (DSC), which detects the heat flow difference between the sample and a reference during heating or cooling, revealing the endothermic or exothermic behavior of the material and the thermal decomposition process [45,47]. The advantage of TG-DSC technology lies in its ability to simultaneously acquire both mass changes and heat flow information, offering a more comprehensive view of the material’s physical and chemical behavior at various temperatures [41,43,46]. TG-DSC precisely characterizes the decomposition temperature and exothermic or endothermic properties of composite materials, helping to determine thermal stability and processing parameters. For hydrogen storage materials, by applying the Kissinger equation (ln(β/T_p_^2^) = ln(AR/E_a_) − E_a_/(RT_p_)), where β is the heating rate (K/min), T_p_ is the desorption peak temperature (K), R is the gas constant (J/mol·K), E_a_ is active energy (kJ/mol), and A is the frequency factor, to fit the TG-DSC results, the activation energy of hydrogen desorption can be analyzed, providing insights into the kinetic properties of the material. Xiao et al. [48] measured the DSC curve of samples and found that after doping MgH_2_ with Ce_0.6_Zr_0.4_O_2_, the desorption peak temperature decreased compared to that of ball-milled MgH_2_ alone (Figure 3a). Two adjacent endothermic peaks appeared on the DSC curve: the first lower-temperature desorption peak was attributed to the activation of MgH_2_ catalyzed by Ce_0.6_Zr_0.4_O_2_, while the second higher-temperature peak was due to non-activated MgH_2_. The Kissinger equation (Figure 3b) was used to calculate the activation energy for the first low-temperature desorption peak of MgH_2_-7CeZrO, which was approximately 66.85 kJ/mol, about 45% lower than that of ball-milled MgH_2_ 121.07 kJ/mol. In addition, the activation energy De-E_a_ of MgH_2_-7CeZrO was 116.06 kJ/mol by fitting the second dehydrogenation peak of MgH_2_-7CeZrO, indicating that De-Ea was close to De-Ea after MgH_2_ grinding, confirming the explanation that the second peak was inactive MgH_2_.

Although TG-DSC is highly effective in analyzing the hydrogen desorption kinetics of hydrogen storage materials, it should be noted that this method cannot monitor the hydrogen absorption process.

### 2.3. Secondary Ion Mass Spectrometry

**Figure 4:**
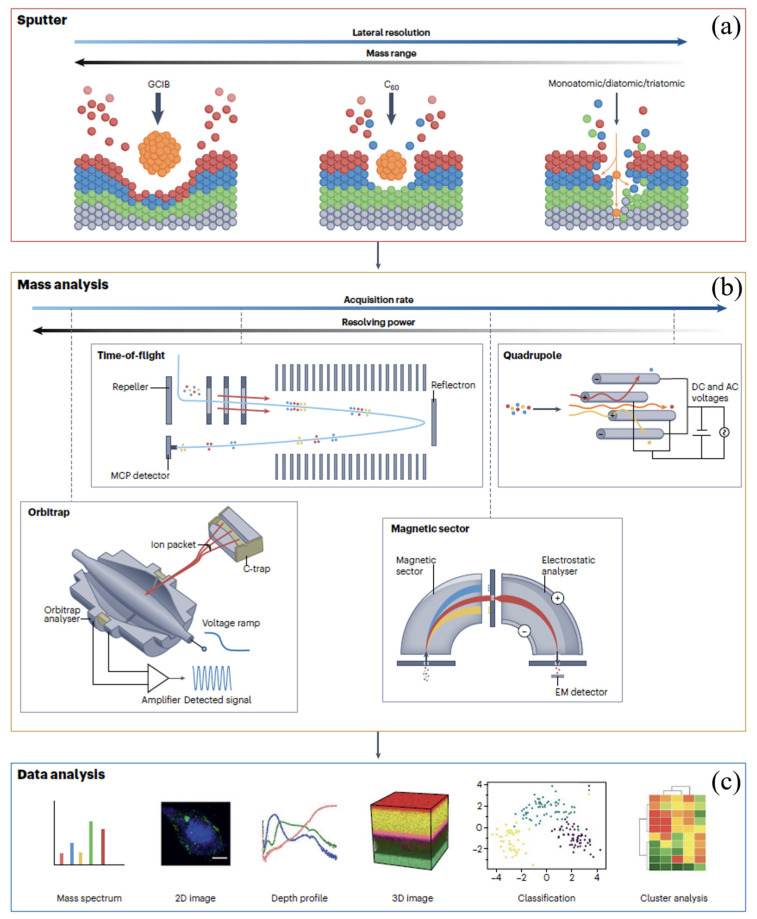
A schematic overview of the secondary ion mass spectrometry experiment [51]. (**a**) A surface is bombarded with a primary ion resulting in the sputtering of secondary ions characteristic of surface chemistry. Secondary ions are detected and measured by mass spectrometry. The bombardment is by primary ions, ranging from atomic ions offering the highest lateral resolution to massive gas cluster ion beams that liberate surface species up to several thousand mass units. (**b**) Mass analysis of secondary ions is generally by quadrupole magnetic sector, time-of-flight, or Orbitrap instruments. (**c**) Outputs from the analysis include mass spectra, 2D or 3D images, and depth profiles, which can be further processed using machine learning. EM, electromagnetic; MCP, microchannel plate [51].

Secondary ion mass spectrometry (SIMS) is a powerful surface characterization technique that provides detailed information about the elemental composition and distribution of hydrogen in storage materials [49,50]. As illustrated in Figure 4, SIMS involves bombarding the sample surface with a high-energy ion beam, producing secondary ions. These secondary ions are separated and detected by a mass spectrometer based on their mass-to-charge ratio (*m/z*), providing information about the composition and structure of the sample surface (Figure 4a). The primary ions (such as Cs^+^ or O^2+^) bombard the sample surface, causing sputtering of the surface material and generating secondary ions [51]. The production rate of secondary ions depends on the surface concentration of the sample and the sputtering yield of the primary ions (Figure 4b). By separating and detecting the secondary ions with a mass spectrometer, the composition and structure of the sample surface elements can be determined (Figure 4c).

**Figure 5:**
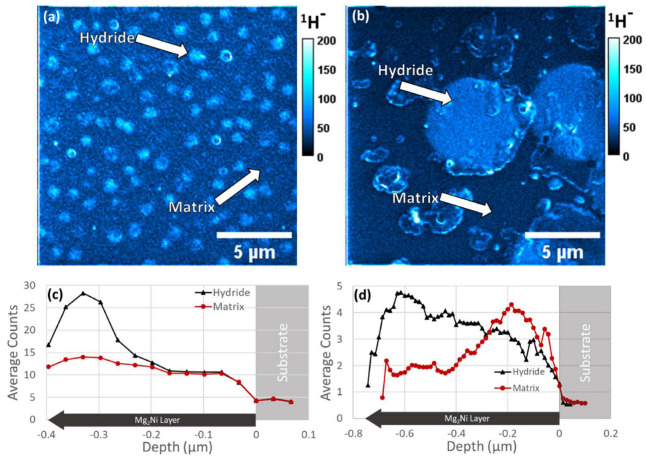
SIMS images from a hydrogenated sample with (**a**) equiaxed microstructure (*Batch A—Sample 1*) showing the summed signal over 3 slices and (**b**) columnar microstructure (*Batch B—Sample 2*) showing the summed signal over 14 slices. SIMS localized depth profiles from regions inside and outside of the surface-visible hydride areas for (**c**) *Sample 1* and (**d**) *Sample 2*. Note that for (**d**), the “hydride” data have been shifted to correct its depth so that both curves agree on the depth of the substrate (zero on the *x*-axis), since the hydrides stick above the surface (discussed below). For (**a**,**c**), 15 total slices were captured with an average slice thickness of 33 nm. Beam current of 50 pA and a dwell time of 4 ms per pixel. Image resolution of 256 × 256 pixels for FOV of 17 × 17 μm. For (**b**,**d**), 56 slices total were collected with an average slice thickness of 14.3 nm. Beam current of 100 pA and a dwell time of 1 ms per pixel. Image resolution of 256 × 256 pixels for FOV of 17 × 17 μm [53].

The different binding energies of hydrogen atoms make the analysis of hydrogen storage processes in carbon-containing materials extremely complex. To differentiate between surface atoms and atoms embedded in the sample, Madroñero et al. [52] used a SIMS spectrometer with periodic ion beam interruption, observing some outgassing phenomena of surface hydrogen under room temperature and high-vacuum conditions. SIMS has proven invaluable in studying the spatial distribution of hydrogen and other elements in complex storage materials. For example, D. Andersen et al. [53] combined SIMS with dual-beam focused ion beam scanning electron microscopy to obtain high-resolution imaging of hydrogen and deuterium in Mg_2_Ni/Mg_2_NiH_4_ hydrogen storage films. This allowed successful characterization of the formation process of hydrides at different depths in the films, providing valuable insights into the hydrogen storage mechanisms of the materials. When the grains exhibit an equiaxed structure (Figure 4c and Figure 5a), hydrides mainly form on the film surface, evidenced by an enhanced ^1^H signal in the surface “hydride” local depth profile. In contrast, when the grains exhibit a columnar structure (Figure 5b,d), the hydrides extend toward the substrate, forming a continuous region. The local depth profile shows that the fully hydride layer is confined near the substrate and is surrounded by a sub-hydride layer.

Although SIMS technology is not extensively employed currently in the analysis of solid-state hydrogen storage materials, its unique advantages, including high spatial resolution, sensitivity to hydrogen concentrations as low as parts per million (ppm), and capability to provide depth profile information, offer significant potential. In the future, SIMS could play a crucial role in supplementing the characterization of hydrogen storage materials, addressing existing gaps in understanding various aspects of these materials [54,55]. To overcome the limitations of SIMS, advanced instrumentation with improved mass resolution and sensitivity has been developed [56]. The use of multi-modal SIMS, combining different primary ion beams and detection modes, has enhanced the capabilities of the technique [57]. Careful sample preparation and the use of appropriate reference materials are essential for accurate quantification [58].

### 2.4. Electrochemical Characterization Methods

Electrochemical methods, including cyclic voltammetry, chronopotentiometry, and electrochemical impedance spectroscopy, are valuable tools for characterizing the electrochemical hydrogen storage properties of materials [59,60]. These techniques provide insights into the charge–discharge behavior, kinetics, and reversibility of hydrogen sorption processes in electrochemical systems, such as metal hydride batteries [61,62,63].

Cyclic voltammetry involves sweeping the potential of the working electrode containing the hydrogen storage material and measuring the resulting current. Chronopotentiometry applies a constant current to the electrode and monitors the potential response over time [64]. Electrochemical impedance spectroscopy (EIS) measures the impedance of the electrochemical system over a wide range of frequencies [65]. To overcome the limitations of electrochemical methods, researchers have developed advanced electrochemical cell designs and measurement protocols. The use of reference electrodes and optimized electrolyte compositions can improve the accuracy and reliability of the measurements [66]. Combining electrochemical methods with other characterization techniques, such as XRD and Raman spectroscopy, can provide a more comprehensive understanding of the electrochemical hydrogen storage behavior. The operational principle of nickel–metal hydride (NiMH) batteries fundamentally involves the absorption and desorption of hydrogen by metal hydrides. This principle can be similarly exploited through electrochemical methods to swiftly assess the performance characteristics of solid-state hydrogen storage materials [67,68,69,70]. Edalati et al. [71] discovered that TixZr_2-x_CrMnFeNi alloys, benefiting from the Ti/Zr ratio of the C14 Laves structure, exhibit good room-temperature hydrogenation/dehydrogenation capabilities. Electrochemical tests on their discharge potential, discharge capacity, and discharge capacity versus cycle number showed that this high-entropy alloy (HEA) successfully functions as the negative electrode of a nickel–metal hydride battery, with excellent charge–discharge cycling performance. The optimal Ti/Zr ratio achieved the highest storage capacity and fastest activation.

## 3. Structure and Composition Characterization Techniques

As the field of hydrogen storage materials evolves, advanced spectroscopic and microscopic techniques have become indispensable for detailed characterization at the molecular and atomic levels [73]. Techniques such as Raman spectroscopy, X-ray diffraction, and X-ray photoelectron spectroscopy offer unparalleled capabilities in probing the structural, electronic, and chemical properties of hydrogen storage materials. These advanced techniques enable researchers to achieve a deeper understanding of the interactions and mechanisms at play within these materials, facilitating the development of more efficient and robust hydrogen storage solutions. By employing these sophisticated methods, scientists can gain comprehensive insights that drive innovation and optimization in the design and application of hydrogen storage materials. Table 2 briefly summarizes the pros and cons of various spectroscopic and microscopic techniques.

### 3.1. Composition Characterization Techniques

#### 3.1.1. Raman and Fourier Transform Infrared Spectroscopy

**Figure 6:**
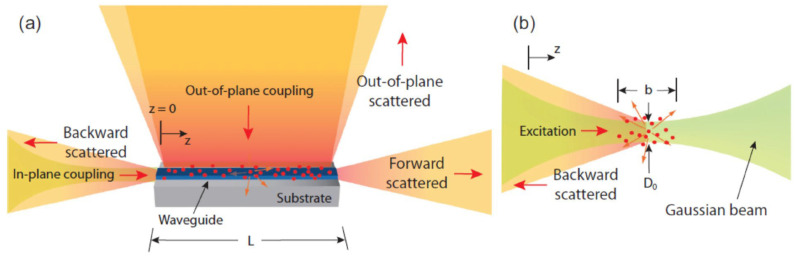
(**a**) Schematic of different configurations of laser excitation and Raman scattered light collection. (**b**) Schematic of laser excitation and Raman scattered light collection in free space [76].

Raman spectroscopy has emerged as a powerful technique for investigating the local structure, bonding, and vibrational properties of hydrogen in storage materials [74,75]. When monochromatic light (usually a laser) illuminates a sample, photons interact with molecules, producing scattered light. A portion of this scattered light undergoes a frequency shift (Raman scattering), which provides information on molecular vibrations and rotations. When the laser illuminates the sample, most photons undergo Rayleigh scattering (elastic scattering with no frequency shift), but a small number of photons undergo Raman scattering (inelastic scattering), with their frequencies shifted due to changes in molecular vibrational or rotational energy levels. Raman-active molecules located near waveguides can be excited through either in-plane coupling (waveguide mode) or out-of-plane coupling (as depicted in Figure 6a). In “classical” Raman scattering, emission typically occurs in the reverse direction to eliminate background interference from the excitation light (as illustrated in Figure 6b). This technique is particularly vital for studying amorphous hydrogen storage materials, providing crucial insights into their structural properties [76].

**Figure 7:**
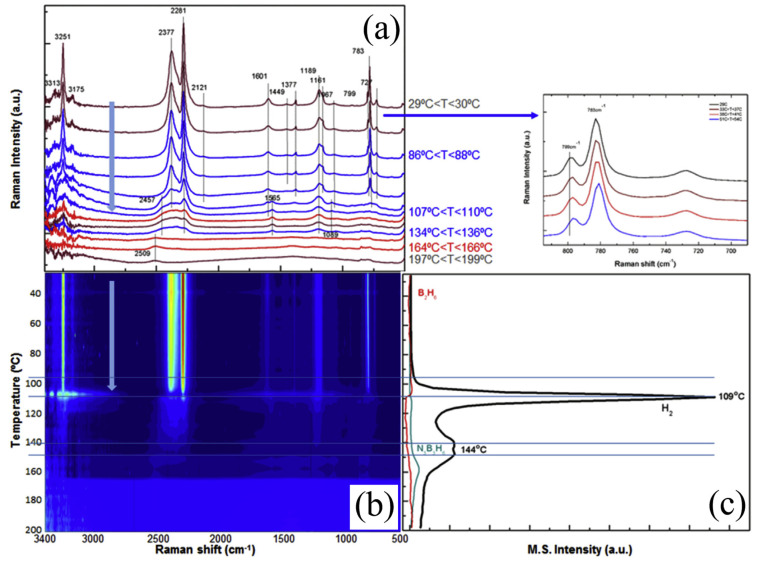
(**a**) Representative Raman spectra. (**b**) In situ Raman profile of materials under heating conditions (from room temperature to 200 °C) and (**c**) simultaneous mass spectrometry profiles for H_2_ and other volatile components evolved during thermal decomposition of neat AB under a ramp of 1 °C·min^−1^ [82].

Raman spectroscopy offers several advantages for characterizing hydrogen storage materials, including its non-destructive and non-contact nature, high spectral resolution, and the ability to identify different hydrogen-bonding configurations [77]. It is particularly useful for studying the interactions between hydrogen and the host material, such as the formation of metal–hydrogen bonds [78]. This technique is particularly vital for studying amorphous hydrogen storage materials, providing crucial insights into their structural properties [79,80,81]. Raman spectroscopy has proven invaluable in studying the local structure and bonding in complex hydrides. Ross et al. [74] used this technique to investigate the decomposition pathway of sodium aluminum hydride (NaAlH_4_), a promising hydrogen storage material. Their study revealed distinct Raman shifts associated with different Al-H bond configurations, providing insights into the dehydrogenation mechanism. Pedraza et al. [82] studied the mechanism of hydrogen release from ammonia borane within mesoporous materials using Raman spectroscopy and mass spectrometry. Figure 6a,b show that, at the point of maximum hydrogen evolution, the deformation mode of -NH_3_ at 1601 cm^−1^ disappears, while two new modes emerge at 1565 cm^−1^ and 1085 cm^−1^, indicating the formation of polymeric aminoborane (PAB). When the temperature reaches around 101 °C, the intensity of these modes decreases significantly, along with other vibrational modes such as B-H, H-B-H, B-N, and N-B-H. At 50 °C, the B-N stretching modes of ^1^⁰B and ^11^B at 799 cm^−1^ and 783 cm^−1^ show a slight redshift (see inset in Figure 7), and around 106 °C, they merge and diminish sharply, almost disappearing at 109 °C. However, the mode near 783 cm^−1^ persists at higher temperatures and is associated with the B-N vibrational mode in polyaminoborane (-[BH₂NH₂]^n−^), indicating the formation of this phase. Additionally, above 100 °C and with Raman shifts higher than 3150 cm^−1^, strong noise appears in the signal. The entire Raman spectrum undergoes significant changes around 106 °C, with all vibrational modes weakening, while hydrogen release becomes highly significant in the online mass spectrometry analysis (Figure 7c).

Fourier transform infrared spectroscopy (FTIR) is an analytical technique that studies the molecular composition and chemical structure of a sample by measuring the absorption or transmission of infrared spectra [89,91]. Different molecular functional groups exhibit specific absorption characteristics for particular wavelengths of infrared light [88,90]. FTIR identifies these characteristic absorption peaks, allowing for rapid, sensitive, and non-destructive qualitative and semi-quantitative analysis [86,87]. It is widely applied in fields such as chemistry, materials science, environmental monitoring, and pharmaceuticals.

**Figure 8:**
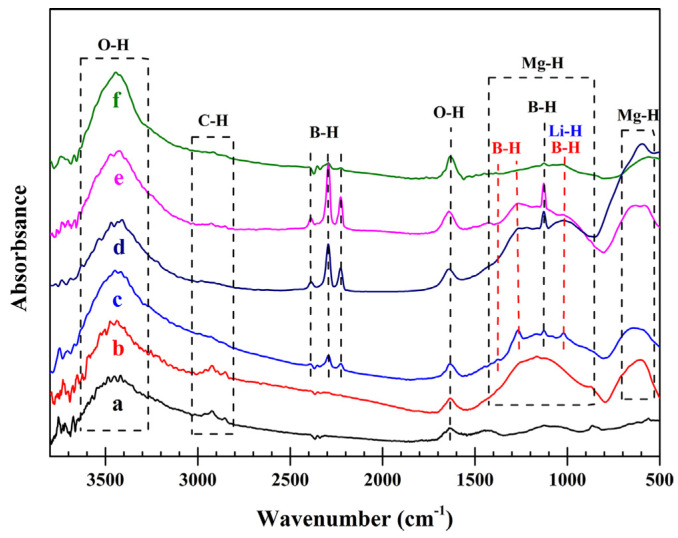
FTIR spectra of (a) the commercially purchased bulk KBr powder, (b) hand-mixed MgH_2_ + 5 vol% C, (c) BMAS powder, (d) BMAS powder after one dehydrogenation (1R) powder, (e) BMAS powder after one dehydrogenation and then re-hydrogenation (1S) powder, and (f) BMAS powder after 7 cycles of dehydrogenation and re-hydrogenation and then dehydrogenation again (8R) powder [37].

In the study of coordination hydrides, FTIR plays a key role as it can accurately detect changes in molecular structure and chemical bonds. Coordination hydrides undergo dynamic changes in metal–hydrogen coordination bonds or hydride groups during hydrogen storage and release processes. FTIR can reveal the mechanisms of hydrogenation and dehydrogenation by monitoring the characteristic absorption peaks of these chemical bonds. By tracking the changes in M–H (metal–hydrogen) bond vibration frequencies, FTIR can directly follow the interactions between metal centers and hydrogen in coordination hydrides during hydrogen absorption and desorption. Different metal coordination centers (e.g., transition metals or rare-earth elements) and hydride combinations produce unique infrared absorption peaks, allowing FTIR to distinguish these changes and identify different hydrogen storage mechanisms. Ding et al. [37] utilized FTIR to investigate the hydrogen storage mechanism of the LiBH_4_-MgH_2_ system prepared via ball milling aerosol spraying (BMAS), as shown in Figure 8. Although the characteristic absorption of LiH at 1030 cm^−1^ overlaps with the absorption band of α-Mg(BH_4_)_2_, the absorption bands of Mg(BH_4_)_2_ at 1262 and 1375 cm^−1^ almost disappeared in the 8R sample, while the absorption band at 1030 cm^−1^ remained visible, indicating the presence of LiH during the reaction process. This observation suggests that Mg(BH_4_)_2_ gradually decomposes over several dehydrogenation cycles, while LiH is formed through the reactions as follows: 12LiBH_4_(s) = Li_2_B_12_H_12_(s) + 10LiH(s) + 13H_2_(g) and Li_2_B_12_H_12_(s) + 6MgH_2_(s) = 6MgB_2_(s) + 2LiH(s) + 11H_2_(g). The gradual increase in the intensity of MgB_2_ and LiH in the samples after cycling reflects the partial reversibility of the above reactions and further explains the gradual decline in hydrogen capacity of the BMAS powders during the cycling process.

Raman spectroscopy is a key tool for studying the hydrogenation and dehydrogenation mechanisms of metal hydrides. By monitoring changes in Raman spectra during hydrogen adsorption, insights into phase transitions, structural changes, and kinetics can be obtained. To fully exploit the potential of Raman spectroscopy, researchers have developed advanced instruments and data analysis methods, such as confocal Raman microscopy for high-resolution spatial mapping and in situ Raman spectroscopy for real-time monitoring of hydrogen adsorption processes. Alongside Raman spectroscopy, FTIR also plays an important role in hydrogen storage studies by detecting changes in chemical bonds between hydrogen and metal or metal oxide matrices, providing molecular vibrational information during hydrogen absorption and desorption.

These two techniques complement each other, with FTIR being particularly advantageous for detecting X-H (such as M-H or O-H) stretching vibrations in hydrides. In combination, Raman and FTIR spectroscopy provide a comprehensive analysis of material structures, chemical bond vibrations, and phase transitions, offering powerful tools for the design and optimization of hydrogen storage materials.

#### 3.1.2. X-Ray Diffraction and Neutron Scattering

**Figure 9:**
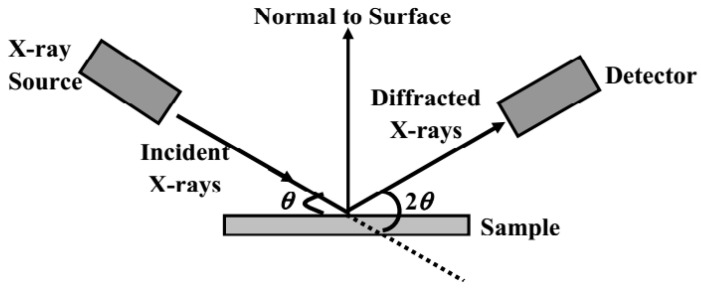
Schematic diagram of the XRD principle.

X-ray diffraction (XRD) is a fundamental technique for characterizing the crystallographic structure, phase composition, and structural changes in hydrogen storage material, and the schematic diagram is shown in Figure 9. Its fundamental equation is Bragg’s law, which describes the conditions for XRD in a crystal. When X-rays illuminate a crystal, the atomic planes within the crystal cause the XRD [92,93]. By measuring the diffraction angles and intensities, one can determine the lattice parameters and atomic arrangement of the crystal [104,105]. Bragg’s law reveals the relationship between the crystal structure and the X-ray wavelength, enabling the inference of the crystal’s three-dimensional structure from its diffraction pattern [103].

**Figure 10:**
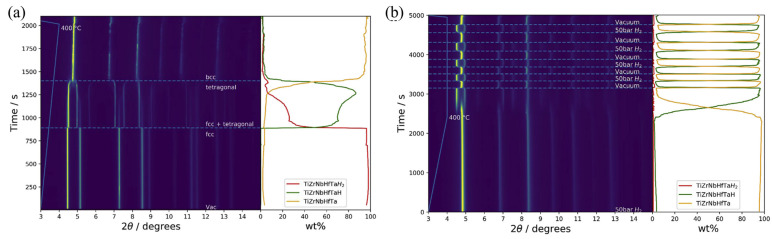
In situ SR-XRD and phase content at 400 °C during (**a**) hydrogen desorption in dynamic vacuum and (**b**) cycling between 50 bar H_2_ and dynamic vacuum [98].

XRD is widely used in hydrogen storage research to investigate the structural properties of metal hydrides, complex hydrides, and other crystalline storage materials [94,95,96]. It allows for the identification of the hydrogen storage phases, the determination of the phase abundances, and the study of phase transitions during the hydrogen sorption processes. One of the advantages of XRD is its non-destructive nature, allowing for the characterization of the bulk properties of the material [97]. It provides statistical information about the average structure, complementing local probe techniques like Raman spectroscopy. In situ XRD has emerged as a powerful tool for studying the structural evolution of hydrogen storage materials during absorption and desorption cycles. Zlotea et al. [98] used in situ XRD to analyze the hydrogen release and absorption process of the TiZrNbHfTa high-entropy alloy. Through in situ XRD (Figure 10a,b), the phase transformations between the alloy, monohydride, and dihydride were observed clearly, greatly aiding researchers in understanding the dynamic hydrogen absorption and desorption processes of the alloy materials.

Furthermore, the advent of high-energy synchrotron X-ray sources has enabled rapid, time-resolved XRD measurements. Jensen et al. [99] leveraged this capability to study the dehydrogenation kinetics of NaAlH_4_, a promising complex hydride. Their millisecond-resolution measurements uncovered transient phases that play a critical role in the hydrogen release process, demonstrating the power of advanced XRD techniques in elucidating complex reaction mechanisms.

**Figure 11:**
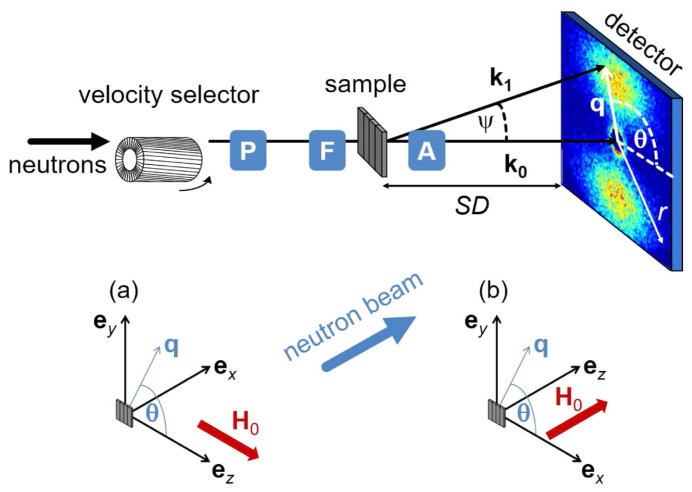
Schematic diagram of a neutron scattering device [102].

Neutron scattering techniques, including neutron diffraction and inelastic neutron scattering, are powerful tools for investigating the structural and dynamic properties of hydrogen in storage materials [100,101]. Neutron scattering involves the interaction of neutrons with the atomic nuclei in the material to study the structure and dynamics of the material. Neutron scattering includes elastic scattering (such as neutron diffraction) and inelastic scattering. When a neutron beam irradiates a sample, neutrons scatter off the sample’s atomic nuclei, and the scattered neutrons are collected by detectors, as illustrated in Figure 11 [102]. By analyzing the angle and energy distribution of the scattered neutrons, information about the atomic structure and dynamics of the sample can be obtained.

**Figure 12:**
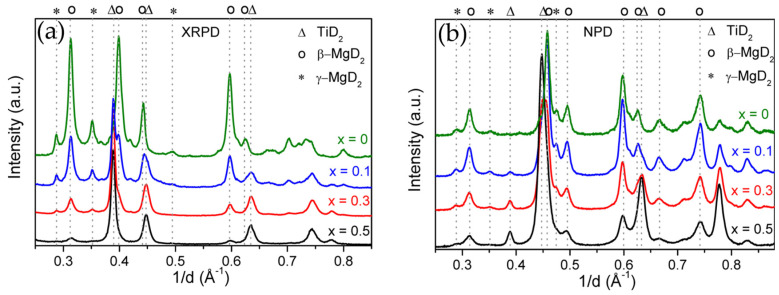
(**a**) X-ray and (**b**) neutron diffraction patterns of deuterated (1−x)MgD_2_−xTiD_2_ nanocomposites for x = 0, 0.1, 0.3, and 0.5 [107].

By measuring and analyzing the scattering cross-section and momentum transfer, the atomic structure and dynamics of the sample can be inferred. Neutrons have unique advantages for studying hydrogen, as they can penetrate deep into the material and have high sensitivity to light elements like hydrogen [106]. In situ neutron scattering can determine the reaction process of Mg-based hydrides by tracking phase changes and distributions during H_2_ desorption and absorption reactions. Ponthieu et al. [107] studied the reversible deuterium absorption of MgD_2_-TiD_2_ nanocomposites using this technique. By examining the in situ H_2_ desorption process, they found that the dehydrogenation peak of 0.3TiH_2_-0.7MgH_2_ appeared at 520 K, approximately 30 K lower than that of pure MgD_2_, and the desorption kinetics were significantly faster. They discovered that the transformation of β-MgD_2_ to Mg is the only reversible loading path for deuterium at moderate pressure and temperature (i.e., *p* < 1 MPa, T < 600 K). The addition of TiD_2_ not only restricted grain growth of the Mg and MgD_2_ phases but also induced lattice distortion in β-MgD_2_. The TiD_2_ phase facilitated hydrogen migration through the sub-stoichiometric MgD_2_-η phase and TiD_2_-η phase, as well as the coherent interface between TiD_2_ and Mg/MgD_2_ phases. As shown in Figure 12a, XRD can clearly characterize the phase composition of the composite material but struggles to distinguish its crystal structure. Therefore, neutron diffraction becomes crucial for analysis. In Figure 12b, the signal intensity of γ-MgD_2_ is significantly stronger than in the XRD results. The combination of these results confirms the coexistence of both β-MgD_2_ and γ-MgD_2_ phases in the composite material. It is worth noting that, in neutron scattering analysis, different hydrogen isotopes may occupy different interstitial sites within the metal lattice and have varying activation diffusion barriers, which could impact the performance analysis of hydrogen storage materials. Thus, ensuring the accuracy of the research is another challenge that must be addressed when using this method in hydrogen storage material studies.

Neutron diffraction provides detailed information about the crystal structure, phase composition, and hydrogen occupancy in storage materials [108,109,110]. Inelastic neutron scattering, on the other hand, probes the vibrational and rotational dynamics of hydrogen within the material [111]. To harness the full potential of neutron scattering techniques, researchers have developed advanced instrumentation and data analysis methods [106]. The use of high-intensity neutron sources and optimized sample environments has enhanced the capabilities of these techniques [112]. Combining neutron scattering with complementary characterization methods, such as XRD and Raman spectroscopy, provides a comprehensive understanding of the structural and dynamic aspects of hydrogen storage materials.

#### 3.1.3. X-Ray Photoelectron Spectroscopy

**Figure 13:**
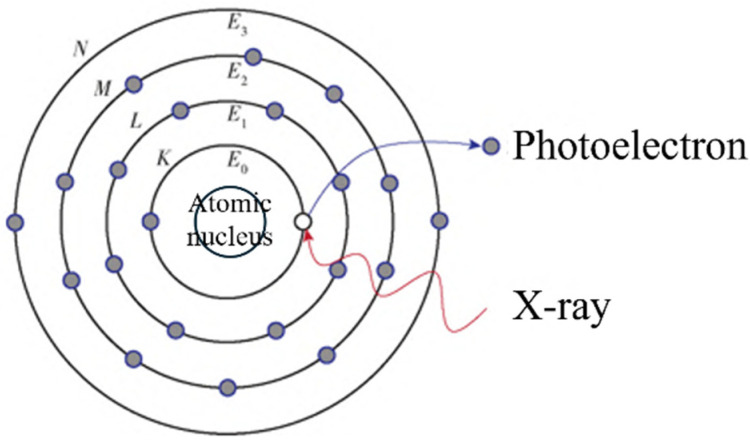
Schematic diagram of the XPS principle. *E* is the binding energy [113].

X-ray photoelectron spectroscopy (XPS) is a surface-sensitive technique that provides valuable information about the elemental composition, chemical states, and electronic structure of hydrogen storage materials [113]. This method is based on the excitation of photoelectrons from a sample by X-rays and the measurement of the photoelectrons’ kinetic energy to determine the elemental composition and chemical states of the sample. When X-rays illuminate a sample, the atoms in the sample absorb the X-ray energy and emit photoelectrons (Figure 13). The kinetic energy of these photoelectrons is related to the energy of the incident X-rays and the binding energy of the atomic nucleus. By analyzing the photoelectron spectrum, one can obtain binding energy information for the elements on the sample surface, thereby determining the sample’s elemental composition and chemical state [114].

XPS is particularly useful for studying the surface chemistry of hydrogen storage materials, as it can probe the top few nanometers of the sample [115]. It can provide insights into the surface oxidation states, contamination levels, and chemical bonding between hydrogen and the host material [116,117]. XPS has been widely used to investigate the surface properties of metal hydrides, complex hydrides, and nanostructured storage materials [118,119].

**Figure 14:**
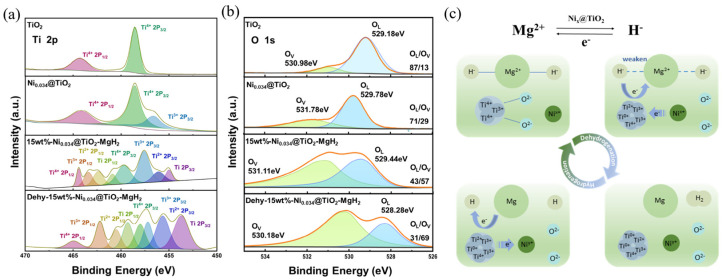
High-resolution XPS spectra of (**a**) Ti 2p and (**b**) O 1s, as well as (**c**) valence changes during the hydrogenation and dehydrogenation processes.

Selvam et al. [120] utilized XPS to analyze Mg_2_Cu and Mg_2_Ni alloys exposed to air, finding that they undergo surface decomposition and preferential segregation of magnesium in the presence of oxygen and moisture. The segregated magnesium primarily existed as oxides and hydroxides on the surface, while Ni or Cu also appeared in oxidized states. The passivation of the alloys was caused by the oxidation of the transition metal components, and the researchers believed that the activation of these alloys involved the reduction of the oxidized three-dimensional elements and the formation of metal clusters. To investigate the influence of TiO_x_ on MgH_2_ in greater depth, Zhang et al. [123] analyzed the internal chemical states of the samples using X-ray photoelectron spectroscopy (XPS). As shown in Figure 14a,b, compared to the single Ti^4+^ state in TiO_2_, the Ti in the Ni_0.034_@TiO_2_ catalyst exhibits a mixed valence state of Ti^4+^ and Ti^3+^. During the dehydrogenation process, the content of Ti^4+^ and Ti^3+^ decreases, while the proportion of Ti^2+^ and Ti^0^ increases. Meanwhile, due to the electronegativity of Ti (1.54), which lies between that of Mg (1.31) and H (2.20), it helps to weaken the Mg-H bond, thereby accelerating the dehydrogenation reaction. Throughout the evolution of Ti valence states, the valence state of oxygen (O) also changes. The O 1s XPS spectra of TiO_2_ show two peaks located at 529.18 eV and 530.98 eV, corresponding to the Ti-O-Ti oxygen lattice (OL) and oxygen vacancies (OVs), respectively. The OL/OV ratio in TiO_2_ is 87/13, while the OL/OV ratio in the Ni_0.034_@TiO_2_ catalyst is 71/29, significantly lower than that of TiO_2_. This indicates that the presence of single-atom Ni promotes the formation of oxygen vacancies. Additionally, the OL/OV ratio in the Nix@TiO_2_ sample is also lower than that in TiO_2_, further proving that Ni facilitates the generation of oxygen vacancies. Combined with X-ray absorption spectroscopy data, the Ni in the Ni_0.034_@TiO_2_ catalyst exhibits a mixed positive valence state, with a strong electron-accepting capability. In this case, Ni attracts O ions, promoting the formation of oxygen vacancies in TiO_2_, resulting in a higher number of oxygen vacancies compared to that of pure TiO_2_. This is also consistent with recent findings on the influence of metal particles on oxygen vacancies. Figure 14c illustrates the catalytic mechanism during hydrogenation and dehydrogenation. Single-atom Ni can promote the formation of OVs and multivalent Ti^x+^ species around TiO_2_ units. Oxygen vacancies serve as active sites that accelerate electron transfer, while Ti^x+^ facilitates transitions between valence states via electron mediation, thus avoiding the high energy required to directly break the Mg-H bond. The atomic interface formed between isolated Ni atoms and Ti^x+^ constitutes dispersed Ni-O-Ti^x+^ active centers, thereby enhancing catalytic performance. Overall, the synergistic interaction between single-atom Ni and the TiO_2_ support significantly improves the catalytic effect.

One of the key applications of XPS in hydrogen storage research is the study of surface catalysts and coatings that enhance the hydrogen sorption kinetics [121,122]. By analyzing the chemical composition and oxidation states of the surface species, the role of catalysts in promoting hydrogen dissociation, diffusion, and recombination can be elucidated. To overcome the limitations of XPS, researchers have developed advanced instrumentation and data analysis methods, such as synchrotron-based XPS for high-resolution measurements and in situ XPS studies for real-time monitoring of surface chemical changes. Combining XPS with other surface characterization techniques, such as scanning tunneling microscopy (STM) and atomic force microscopy (AFM), has provided a comprehensive understanding of the surface morphology and chemical properties of hydrogen storage materials. XPS has been crucial in understanding surface phenomena in hydrogen storage materials, particularly catalytic effects and degradation mechanisms.

### 3.2. Structure Characterization Techniques

#### 3.2.1. Scanning Electron Microscopy

Scanning electron microscopy (SEM) is an ideal tool for studying the microstructure and surface characteristics of hydrogen storage materials due to its high-resolution imaging capabilities [125]. SEM can reveal detailed morphological features of materials, helping scientists understand the interactions between hydrogen and these materials, which is crucial for designing more efficient hydrogen storage systems [126,127]. The microstructure of materials, such as pore size, distribution, and surface roughness, directly affects the adsorption and diffusion rates of hydrogen.

**Figure 15:**
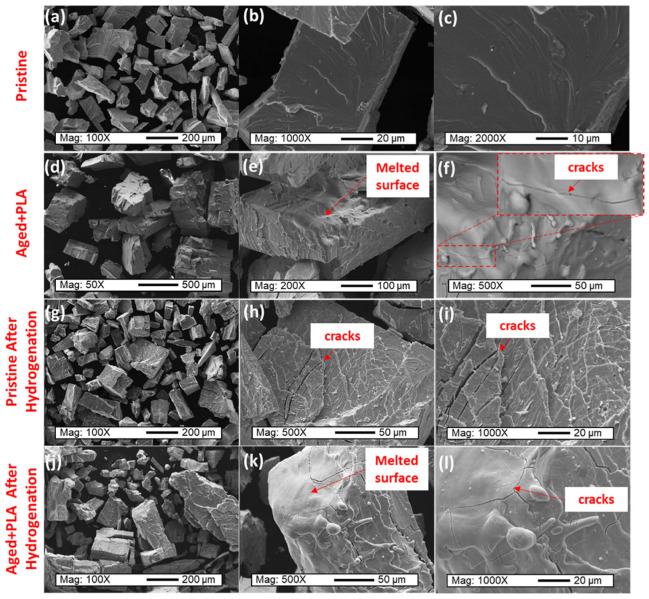
SEM analyses of the (**a**–**c**) pristine sample, (**d**–**f**) aged + PLA sample, (**g**–**i**) pristine sample after hydrogenation, and (**j**–**l**) aged + PLA sample after hydrogenation [124].

Through SEM, researchers can clearly see these structural features and evaluate their specific impact on hydrogen storage performance. For example, larger pores may promote rapid hydrogen diffusion, while higher surface roughness can increase the surface area, providing more active sites for hydrogen adsorption. Additionally, SEM analysis can reveal potential defects on material surfaces, such as cracks, fractures, or other irregular shapes, which could affect the long-term stability and hydrogen storage efficiency of the materials. By regularly using SEM to monitor these materials, scientists can track performance changes during long-term use and adjust preparation processes or select more suitable materials accordingly. Silva et al. [124] used SEM to observe the surface morphology of Ti_11_V_30_Nb_28_Cr_31_ at different stages of hydrogenation, finding that during laser processing, the surface of the debris particles melted, increasing the proportion of oxides near the surface. The manual grinding process leads to random particle size distribution, as shown in Figure 15a,d,g,j. Figure 15b,c illustrate that the surface of the particles after breaking the original alloy remains smooth with sharp edges, consistent with the brittle characteristics of the alloy. Figure 15e,f show that laser treatment significantly alters the particle surface, where rounded edges and a smooth surface suggest that the particles underwent remelting and rapid solidification. The inset in Figure 15f reveals microcracks on the remelted surface, which may contribute to the activation of the sample. Additionally, the remelted surface could restore the hydrogenation ability of the aged sample. Figure 15h,i,k,l display the similar behavior of both original and aged samples during hydrogenation. Surface cracks caused by volume expansion during the hydrogenation process were observed in particles analyzed under both conditions. These changes enhanced the alloy’s hydrogen storage capacity. Therefore, surface remelting, oxide layer formation, and crack formation were confirmed to be factors influencing the hydrogen storage capacity of the pulse laser-activated Ti_11_V_30_Nb_28_Cr_3_1 alloy.

In recent years, the development of environmental scanning electron microscopy (ESEM) has brought revolutionary advancements to hydrogen storage research [128,131]. Unlike traditional SEM, ESEM allows for sample observation under near-natural conditions without requiring high vacuum or complex sample preparation [130,132]. This enables researchers to directly monitor and record changes in material surfaces and microstructures during hydrogen absorption and desorption processes in real time. ESEM is particularly suitable for studying the interactions between hydrogen and materials. During hydrogen absorption, ESEM can capture morphological changes on the material surface, such as surface expansion, crack formation, or other structural deformations, in real time. These observations provide valuable information for optimizing material design and improving reaction speed and hydrogen storage capacity. Similarly, during hydrogen release, ESEM can offer crucial visual evidence to help scientists understand the material’s regeneration capability and long-term stability.

#### 3.2.2. Transmission and Scanning Transmission Electron Microscopy

Advanced electron microscopy techniques, particularly transmission electron microscopy (TEM), have revolutionized our understanding of hydrogen storage materials at the atomic scale [140]. With its superior resolution and accuracy, TEM allows researchers to observe the atomic and molecular structure of materials in unprecedented detail. This unique perspective provides scientists with critical insights into how these materials behave during hydrogen storage [142].

**Figure 16:**
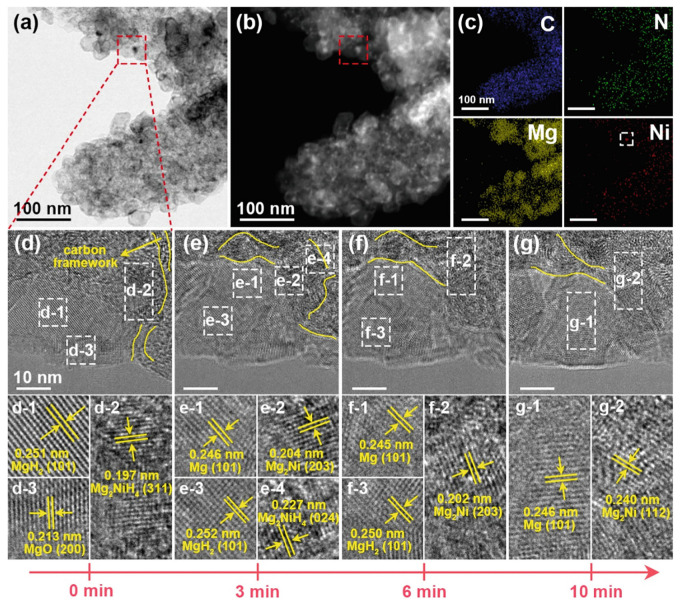
In situ TEM analysis of the hydrogenated MgH_2_/Ni@pCNF composites: (**a**) HAADF image (the square marked by red dotted line indicates the irradiated area). (**b**) BF image. (**c**) The corresponding elemental mapping of C, N, Mg, and Ni. (**d**–**g**) HRTEM images and selective electron diffraction at random points showing the evolution of microstructure upon hydrogen desorption induced by the electron-beam irradiation. (**d1**–**d3**) Initial microstructure showing lattice fringes of MgH_2_ (101), Mg_2_NiH_4_ (311), and MgO (200), respectively, before irradiation. (**e1**–**e4**) After 3 min, partial decomposition of Mg_2_NiH_4_ into Mg_2_Ni begins, with defects forming at the Mg_2_NiH_4_/MgH_2_ interface, promoting hydrogen desorption, while some MgH_2_ remains stable. (**f1**–**f3**) At 6 min, complete decomposition of Mg_2_NiH_4_ is observed, while MgH_2_ remains partially stable, and Mg nanoparticles become visible. (**g1**,**g2**) After 10 min, hydrogen is fully released and transferred to Mg and Mg_2_Ni [133].

Through TEM, scientists can directly observe the atomic arrangement and molecular configuration within materials [141]. This capability not only helps reveal the fundamental structural characteristics of materials but also allows researchers to see subtle changes in the internal structure during hydrogen adsorption and desorption [143]. For example, researchers can observe how hydrogen atoms bond with specific sites within the material or how the lattice structure of the material deforms during hydrogen absorption. These detailed observations provide valuable information for understanding the behavior of hydrogen storage materials. By analyzing these microstructural changes, scientists can better comprehend the mechanisms of hydrogen adsorption and the key factors influencing storage capacity and release rate [149]. This in-depth understanding aids in developing new materials and optimizing the chemical composition and microstructure of existing materials to enhance their hydrogen storage performance. Furthermore, TEM’s high-resolution imaging allows researchers to identify small defects within materials, such as dislocations, vacancies, and interfacial mismatches. These defects significantly impact the overall performance of materials, especially during repeated cycles of hydrogen adsorption and desorption. Therefore, accurately identifying and analyzing these defects is crucial for designing more durable and efficient hydrogen storage materials. Wu et al. [150] prepared LiBH_4_ composites confined within bilayer carbon nanobowls through a strong capillary effect under 100 bar H_2_ pressure. TEM analysis confirmed the gradual formation of bilayer carbon nanobowls. Benefiting from the nanoscale confinement and catalytic functions of carbon, the composite released hydrogen from 225 °C, peaking at 353 °C, with a hydrogen release amount of up to 10.9 wt.%. Compared to bulk LiBH_4_, the peak dehydrogenation temperature decreased by 112 °C. More importantly, the composite absorbed about 8.5 wt.% H_2_ at 300 °C and 100 bar H_2_, demonstrating significant reversible hydrogen storage capability. Ren et al. [133] investigated the dehydrogenation mechanism of the MgH_2_/Ni@pCNF composite using in situ high-resolution transmission electron microscopy (HRTEM) to observe the microstructural evolution under electron-beam irradiation. Figure 16a–c show HAADF, BF, and corresponding element mapping images of hydrogenated MgH_2_/Ni@pCNF, with the red dashed box indicating the irradiated area. Figure 16d–g present the HRTEM images of the composite material during the hydrogen release process, where the lattice fringes observed in the selected area electron-diffraction patterns in each subfigure correspond to the phase changes of the material throughout the reaction. Before irradiation, lattice fringes were used to identify MgH_2_ (101) (Figure 16(d1)), Mg_2_NiH_4_ (311) (Figure 16(d2)), and MgO (200) (Figure 16(d3)). Additionally, amorphous carbon frameworks of pCNF, acting as scaffolds for the nanoconfined MgH_2_, were observed in all HRTEM images (Figure 16d–g). After 3 min of irradiation, part of the Mg_2_NiH_4_ began to decompose, converting into Mg_2_Ni (Figure 16(e2)). A 0.246 nm plane spacing was observed between MgH_2_ (Figure 16(e3)) and Mg_2_NiH_4_ (Figure 16(e4)), corresponding to Mg (101) (Figure 16(e1)), indicating that MgH_2_ near MgH_2_NiH₄ was also starting to decompose. The desorption of Mg_2_NiH_4_ induced lattice volume changes, which introduced internal stress and defects at the Mg_2_NiH_4_/MgH_2_ interface, promoting MgH_2_ desorption. Furthermore, the interface between the catalyst (Mg_2_NiH_4_/Mg_2_Ni) and the matrix (MgH_2_) facilitated rapid hydrogen diffusion, accelerating MgH_2_ desorption. After 6 min of irradiation, only Mg_2_Nif (Figure 16(f2)), Mg (Figure 16(f1)), and MgH_2_ (Figure 16(f3)) remained, indicating that Mg_2_NiH_4_ completely decomposed earlier than MgH_2._ After 10 min of electron-beam irradiation, the hydrogen in the irradiated area was fully released and transferred to Mg (Figure 15(g1)) and Mg_2_Ni (Figure 15(g2)). Moreover, due to the confinement of pCNF, the Mg-based nanoparticles did not experience significant growth or agglomeration.

Additionally, the development of in situ environmental transmission electron microscopy (E-TEM) has made it possible to observe materials directly under dynamic, real-world conditions, which is crucial for studying hydrogen storage materials [147,148]. Traditional TEM requires vacuum conditions, limiting the observation of material behavior under actual operating conditions [145,146]. In contrast, E-TEM allows for the observation of materials in a gaseous environment, which can include hydrogen, thus providing genuine insights into the behavior of these materials during hydrogen adsorption and desorption [144]. Through E-TEM, researchers can observe structural changes during the hydrogen cycling process in real time [151,152]. This includes observing how atoms rearrange, how defects in the material evolve, and how the crystal structure of the material changes during hydrogen adsorption and release. These observations are critical for understanding the mechanisms of hydrogen adsorption and the factors influencing the efficiency and durability of storage materials. Future rational use of E-TEM can help identify the best materials and designs for hydrogen storage, allowing scientists to conduct experiments on different materials and under various environmental conditions.

#### 3.2.3. Atomic Force Microscopy

Atomic force microscopy (AFM), as a precise surface analysis tool, has provided valuable insights into the surface morphology and mechanical properties of hydrogen storage materials [135,137]. AFM measures forces through interactions between the probe and the sample surface, allowing for nanoscale mapping of material surfaces [136,138]. This detailed surface characterization is crucial for understanding and optimizing the performance of hydrogen storage materials [134]. AFM’s high-resolution imaging capabilities enable it to reveal the microstructure of materials, such as nanoparticles, pores, and cracks, which are key factors in evaluating the adsorption capacity of materials. Furthermore, AFM can measure mechanical properties such as hardness and elastic modulus, which are critical for designing hydrogen storage systems that maintain structural stability under various operating conditions.

**Figure 17:**
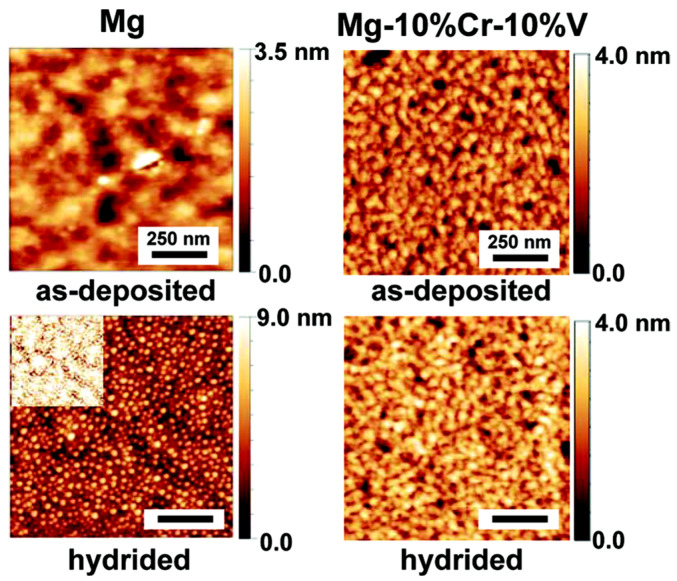
AFM micrographs of Ta/Mg/CrV/Pd and Ta/Mg-10%Cr-10%V/CrV/Pd in the as-deposited and hydrogenated state. The Ta/Mg/CrV/Pd was hydrogenated at 50 mbar for 14 h and Ta/Mg-10%Cr-10%V/CrV/Pd at 10 mbar for 20 h. The inset shows the micrograph of the hydrided film on the same brightness scale as the as-deposited state for Ta/Mg/CrV/Pd [139].

Kalisvaart et al. [139] used AFM to analyze the surface changes of Mg and Mg-10%Cr-10%V films in both deposited and hydrogenated states. As shown in Figure 17, the surface of the deposited palladium (Pd) film is extremely smooth, with a root-mean-square (RMS) roughness of only 5 Å. In the hydrogenated state, the Pd layer appears to break into small particles with diameters of approximately 20 nm, leading to a 2- to 13-fold increase in the measured RMS roughness. Due to the tip effect, atomic force microscopy (AFM) often underestimates roughness. In fact, because of the close spacing of Pd particles, the relatively large tip radius of the AFM (6 nm) almost certainly leads to a significant underestimation of roughness, especially in hydrogenated samples. Therefore, the increase in surface roughness observed after combining neutron reflectometry (NR) data are primarily attributed to the fragmentation of the Pd layer into small particles.

Recent advancements in high-speed atomic force microscopy (high-speed AFM) technology have enabled scientists to observe dynamic processes at the nanoscale in real time [153,154]. High-speed AFM significantly improves imaging speed, allowing researchers to observe and record changes in material surfaces during hydrogen adsorption and desorption almost in real time [155,156]. This capability is particularly important for understanding the dynamic characteristics of hydrogen–material interactions [157]. For example, through high-speed AFM, researchers can directly observe changes in surface morphology caused by hydrogen adsorption, such as slight expansion or contraction of the surface. These changes might be difficult to capture with traditional AFM due to their rapid occurrence. Additionally, this technology can be used to study the fatigue behavior of materials during multiple cycles of hydrogen adsorption and desorption, providing direct experimental data for assessing the long-term stability and reusability of materials.

## 4. Challenges and Limitations

### 4.1. Obstacles and Limitations in Hydrogen Storage Performance Characterization

The characterization of hydrogen storage performance faces multifaceted challenges that significantly impact the accuracy, reliability, and interpretability of experimental data.

Volumetric measurements, particularly the Sieverts method, are susceptible to systematic errors arising from thermal gradients, pressure sensor drift, and gas impurities. Zhou et al. [21] highlighted the critical impact of temperature gradients on volumetric measurements, demonstrating how even minor thermal fluctuations can lead to substantial errors in calculated hydrogen uptake. This issue is particularly pronounced for materials with low storage capacities or slow kinetics, where small measurement errors can lead to significant overestimation or underestimation of storage performance.

The challenge of achieving true equilibrium conditions during measurements is exacerbated by the slow kinetics of many advanced storage materials. Complex hydrides and nanostructured composites often exhibit multi-step absorption/desorption processes with varying time scales, making it difficult to determine when true equilibrium has been reached. This kinetic limitation can lead to underestimation of storage capacities and misinterpretation of thermodynamic parameters, particularly when fixed measurement times are used across different materials.

The discrepancy between laboratory-scale measurements and real-world performance remains a significant hurdle. Factors such as heat and mass transfer limitations, which are often negligible in small-scale experiments, become critical in larger systems. The work of Ding et al. [28] on nanostructured LiBH_4_-MgH_2_ systems exemplifies this challenge, where the excellent performance observed in laboratory tests may not directly translate to practical storage systems due to scaling effects on heat transfer and reaction kinetics.

### 4.2. Challenges and Constraints in Structure and Composition Characterization

The structural and compositional characterization of hydrogen storage materials presents unique challenges that limit our ability to fully understand storage mechanisms and material properties.

In situ characterization, which observes materials in their “native environment”, and operando characterization, which captures real-time data under “actual working conditions”, can provide researchers with deep insights into the structural and functional changes of materials. Although in situ and operando characterization techniques are highly powerful, they often require certain compromises in experimental conditions, such as reduced resolution, decreased sensitivity, or simplified setups, to meet the demands of real-time monitoring. In situ TEM, for instance, allows real-time observation of structural changes during hydrogen absorption/desorption but typically operates at lower pressures than those used in practical storage systems. This pressure gap can lead to observations that may not accurately represent material behavior under realistic conditions. The study by Ren et al. [133] on MgH_2_/Ni@pCNF composites using in situ HRTEM illustrates both the power and limitations of these techniques in studying the dehydrogenation mechanism of complex nanostructured materials.

Raman spectroscopy, while sensitive to hydrogen-containing bonds, faces challenges in quantitative analysis due to variations in scattering cross-sections and the potential for laser-induced sample heating. The work of Pedraza et al. [82] on ammonia borane decomposition demonstrates both the power and limitations of Raman spectroscopy in studying hydrogen storage materials, highlighting the need for careful experimental design and data interpretation.

The characterization of multi-component and nanostructured materials presents additional complexities. Techniques like XPS and SIMS offer high surface sensitivity but may not accurately represent bulk compositions. Conversely, bulk techniques may overlook critical surface phenomena that govern hydrogen uptake and release. The study by Xing et al. [121] on carbon-coated CoNi nanocatalysts illustrates the challenge of characterizing complex nanostructured materials, where the distribution and chemical state of catalytic components play crucial roles in enhancing storage performance.

Addressing these challenges requires continued development of advanced characterization tools, improved experimental protocols, and sophisticated data analysis methods. Emerging approaches, such as machine learning-assisted data interpretation and multi-modal characterization platforms [158], offer promising avenues for overcoming current limitations. However, realizing the full potential of these advanced characterization approaches will require close collaboration between experimentalists, theorists, and instrument developers to ensure that the data obtained accurately reflects the intrinsic properties and behavior of hydrogen storage materials under realistic operating conditions.

## 5. Conclusions and Perspective

The field of solid-state hydrogen storage materials has made significant strides in recent years, with the development of advanced characterization techniques and the emergence of novel materials. However, ongoing challenges in understanding the complex hydrogen storage mechanisms and optimizing material performance necessitate continued research and innovation.

This comprehensive review has provided an overview of the key characterization techniques employed in the field of solid-state hydrogen storage, discussing their principles, advantages, limitations, and synergistic applications. Conventional techniques such as Sieverts method, gravimetric analysis, SIMS, TDS, neutron scattering, and electrochemical methods have been discussed in detail, highlighting their roles in unraveling the intricate relationship between the structure, composition, and properties of hydrogen storage materials. Emerging optical characterization techniques, including Raman spectroscopy, XRD, and XPS, have been explored, emphasizing their potential in providing insights into the local structure, bonding, and surface chemistry of these materials.

Practical considerations, such as equipment availability, sample preparation, and cost-effectiveness, have been addressed to provide a pragmatic guide for researchers in the field. The challenges associated with characterizing novel hydrogen storage materials, such as nanoconfined hydrides, MOFs, and graphene-related materials, have been highlighted, and innovative approaches to tackle these challenges have been discussed.

Looking ahead, the integration of in situ and operando characterization techniques, computational modeling, and data-driven approaches will be crucial for accelerating the discovery and optimization of high-performance hydrogen storage materials. Collaborative efforts among researchers from diverse disciplines and the establishment of standardized characterization protocols and databases will be essential for advancing the field towards practical applications.

As the world transitions towards a sustainable energy future, the development of efficient and reliable hydrogen storage solutions will play a critical role in enabling the widespread adoption of clean energy technologies. By addressing the characterization challenges and embracing innovative approaches, the scientific community can unlock the full potential of solid-state hydrogen storage materials and contribute to the realization of a hydrogen-based energy economy.

## Figures and Tables

**Table 1:** The advantages and limitations of essential characterization techniques.

Method	Advantages	Limitations	Refs.
Sieverts method	Simplicity and reliability, measures hydrogen sorption isotherms, provides thermodynamic information	Sensitivity to volume calibration and leaks, limited information on kinetics, requires accurate temperature control	[26,30,32,33,34,35,36,37,38,39,40]
Gravimetric analysis	High sensitivity, real-time monitoring of hydrogen sorption, enables kinetic studies	Sensitivity to buoyancy effects, influence of impurities and adsorbed species, requires careful calibration and correction procedures	[39,40]
Thermogravimetric analysis Differential scanning calorimetry	Simultaneously measure changes in mass and heat flow, providing comprehensive information about thermal stability, decomposition, and phase transitions of materials in a single experiment	Limited sensitivity in detecting small mass changes and subtle thermal events, making them less suitable for analyzing materials with very low levels of thermal degradation or phase change	[41,42,43,44,45,46,47,48]
Secondary ion mass spectrometry	High spatial resolution, sensitive to low hydrogen concentrations, provides depth profiling information	Destructive technique, requires careful calibration for quantitative analysis, challenging data interpretation due to matrix effects	[49,50,51,52,53,54,55,56,57,58]
Electrochemical methods	Simulates real-world operating conditions, provides information on charge–discharge behavior and kinetics, enables the study of cycle stability and long-term performance	Sensitivity to electrode preparation and cell configuration, requires careful interpretation of electrochemical data, may not provide direct structural and chemical information	[59,60,61,62,63,64,65,66,67,68,69,70,71]

**Table 2:** The advantages and limitations of advanced spectroscopic and microscopic techniques.

Method	Advantages	Limitations	Refs.
Raman spectroscopy	Non-destructive and non-contact technique, high spectral resolution, identifies different hydrogen-bonding configurations	Sensitivity to sample surface and orientation, challenging interpretation for complex materials, may not provide quantitative hydrogen content information	[74,75,76,77,78,79,80,81,82,83,84,85]
Fourier transform infrared spectroscopy	Rapid, non-destructive detection with high sensitivity to low-concentration molecular vibrations; wide range of organic and inorganic materials; excels in identifying functional groups and chemical bonds	It is sensitive to moisture, with water absorption peaks potentially interfering with analysis. It only detects infrared-active functional groups, making non-polar bond vibrations difficult to observe.	[37,86,87,88,89,90,91]
X-ray diffraction	Crystalline Structure Determination, wide range of materials, Phase Identification	Limited to Crystalline Materials, Penetration Depth, Size Limitation	[92,93,94,95,96,97,98,99,100,101,102,103,104,105]
Neutron scattering techniques	Non-destructive technique, provides bulk structural and dynamic information, sensitive to light elements like hydrogen	Requires access to specialized neutron sources, complex data interpretation, challenging sample preparation	[106,107,108,109,110,111,112]
X-ray photoelectron spectroscopy	Surface-sensitive technique, provides elemental composition and chemical state information, investigates surface catalysts and coatings	Limited information on bulk properties, requires clean and well-defined sample surface, may not provide direct hydrogen content information	[113,114,115,116,117,118,119,120,121,122,123]
Scanning electron microscopy	High-resolution surface imaging, large depth of focus, suitable for three-dimensional topography observation, simple sample preparation	Can only observe surface structures; cannot provide internal structural information; may require metal coating, which affects the true morphology	[124,125,126,127,128,129,130,131,132]
Atomic force microscopy	Ultra-high resolution, reaching atomic level; does not require a vacuum environment, allowing for observation of live samples; capable of measuring mechanical and electrical properties of materials	Slow scanning speed, suitable for small area samples; influenced by probe shape, which may cause artifacts; requires surface flattening treatment of the sample	[133,134,135,136,137,138]
Transmission and scanning transmission electron microscopy	Extremely high resolution, capable of observing atomic-level structures; can provide internal structural information of samples; able to perform compositional and phase analysis	Samples must be very thin; complex sample preparation, which may introduce artifacts; requires a vacuum environment, potentially causing sample damage	[139,140,141,142,143,144,145,146,147,148]
